# Supplementary material for: Structural Insights into Cir-mediated Killing by the Antimicrobial Protein Microcin V
Source: bioRxiv. 2025 Mar 9:2025.03.06.641670. Preprint. [Version 1] doi: 10.1101/2025.03.06.641670 (PMC12190408; doi:10.1101/2025.03.06.641670)
Supplement: Supplement 2 [file media-2.pdf]

Table 1: Cryo-EM Data Collection and Structure Statistics

	Cir/MccV
Data Collection	
Magnification	105,000x
Voltage (keV)	300
Total Dose (e/Å^2)	59.65
Number of Frames	30
Exposure Time (s/frame)	0.075
Defocus Range (μm)	-0.8 to -2.4
Pixel Size (Å)	0.83
Image Processing	
Movies Collected	7,606
Micrographs Selected	5,960
Particles Extracted	7,726,727
Final Map Particles	102,419
Symmetry Imposed	C1
FSC Threshold	0.143
Final Map Resolution (Å)	2.9
Resolution Range (Å)	3.06 – 2.71
Atomic Model	
Protein Residues	664
Chains	2
Validation	
Ramachandran Favored (%)	97.73
Ramachandran Allowed (%)	2.27
Ramachandran Outliers (%)	0
Rotamer Outliers (%)	0
RMSD Bond Lengths (Å) (# > 4σ)	0.002 (0)
RMSD Bond Angles (°) (# > 4σ)	0.419 (0)
Clash Score	5.60
Map CC (mask)	0.87
Map CC (volume)	0.88
Deposition IDs	
PBD	9NN6
EMDB	EMD-49565
